# The dual structure of anxiety: reconciling general and specific approaches

**DOI:** 10.3389/fpsyg.2026.1774838

**Published:** 2026-05-08

**Authors:** Evgeniia Alenina, Vladimir Kosonogov

**Affiliations:** Affective Psychophysiology Laboratory, Institute of Health Psychology, HSE University, Saint Petersburg, Russia

**Keywords:** anxiety, anxiety modeling, dual structure, specific anxiety, trait anxiety

## 1 Introduction

Anxiety is a predisposition to worry that manifests across different domains. It has been described as a stable individual trait (e.g., trait anxiety; Spielberger, 1980), current emotional responses (state anxiety; Spielberger, 1983), a clinical disorder [e.g., Generalized Anxiety Disorder (GAD); Spitzer et al., 2006], and domain-specific worries such as math anxiety (Dowker et al., 2016), social anxiety (Telch et al., 2004), or spatial anxiety (Lawton, 1994), among others. However, it remains unclear whether domain-specific anxieties are manifestations of general anxiety or distinct phenomena. Trait anxiety reflects a stable tendency to experience worry without specific stimuli. In contrast, generalized anxiety disorder (GAD) is marked by uncontrollable worry, ruminative thoughts, and tension, largely aligning with the DSM-5 criteria (American Psychiatric Association, 2013). At the same time, thresholds for clinical and non-clinical manifestations remain debatable. While domain-specific anxieties (e.g., math anxiety) are usually assessed with self-report questionnaires targeting worry in specific contexts (e.g., during math tasks), no single instrument captures both general and specific anxiety dimensions simultaneously.

Current literature describes anxiety from different perspectives but not as a unified construct within studies. Different forms of anxiety can be distinguished along several conceptual dimensions that are critical for model specification. First, in terms of temporal dynamics, current literature differentiates state anxiety (a transient, situation-bound arousal linked to salience network activation) from trait anxiety (a stable predisposition associated with enduring alterations in default-mode connectivity; Saviola et al., 2020). Second, regarding the object of response, fear represents a present-focused reaction to immediate threat with strong autonomic mobilization, whereas anxiety is future-oriented, diffuse, and cognitively mediated; although the Research Domain Criteria (RDoC) framework distinguishes “acute” vs. “potential” threat, empirical distinctions in behavior and circuitry often overlap (Ohi et al., 2025). Third, at the clinical level, anxiety disorders (e.g., GAD, panic disorder, specific phobias) share core features of excessive, uncontrollable worry and avoidance but differ in temporal dynamics and trigger specificity. For instance, GAD involves chronic, free-floating worry across multiple domains, whereas phobias are cued by discrete stimuli (Craske and Stein, 2016). Finally, the boundary between normative worry and clinical pathology rests less on symptom content than on persistence, perceived uncontrollability, and functional impairment.

These conceptual distinctions map onto, but are not identical with, statistical frameworks used to model anxiety structure. Hierarchical models posit a broad general distress factor (aligned with trait anxiety/neuroticism) that accounts for covariation among domain-specific dimensions (e.g., panic, social anxiety; Zinbarg and Barlow, 1996). Bifactor models extend this by specifying a general factor plus orthogonal specific factors, allowing researchers to divide variance into shared vs. unique components (e.g., Sexton et al., 2003), a feature that aligns well with our proposal that domain-specific anxieties retain predictive power beyond general predisposition. Spectrum approaches (e.g., Watson, 2005) further contextualize anxiety within broader internalizing spectra, supporting transdiagnostic assessment.

Our dual-structure model integrates these perspectives: it adopts the hierarchical predisposition of a general vulnerability factor but theorizes that domain-specific residuals are not mere statistical artifacts, they reflect specific worry systems that selectively impair performance in specific domains. This moves beyond purely statistical bifactor modeling toward an account of how general and specific anxieties interact.

We evaluate three interconnected research questions: (1) at what level general anxiety transforms into a clinical disorder and whether this threshold is domain-invariant; (2) whether domain-specific anxieties manifest only at high levels of general anxiety or represent separate psychological constructs; and (3) whether a single tool could cover most anxiety manifestations (both general and specific).

### 1.1 General(ised) anxiety

Trait anxiety (TA) and GAD are typically treated as distinct categories: a personality trait vs. a clinical diagnosis. However, this boundary may be vague. Trait anxiety, as defined by Spielberger (1980), reflects a stable tendency to worry even without external stimuli. It is usually measured by questionnaires assessing general feelings, including items on self-doubt and helplessness. In contrast, GAD is a clinical diagnosis that requires persistent, excessive, and uncontrollable worry, along with symptoms such as restlessness, fatigue, irritability, or sleep problems causing significant distress for over 6 months (American Psychiatric Association, 2013).

At first glance, the distinction seems clear: TA represents a tendency to worry, whereas GAD denotes a disorder with uncontrollable worry. This distinction between them rests less on symptom content rather than on perceived controllability and functional impact (e.g., “I wish I could be as happy as others seem to be” for trait anxiety (Spielberger, 1983) and “Not being able to stop or control worrying” (Spitzer et al., 2006). Individuals high in TA may worry frequently yet retain regulatory flexibility; in GAD, worry persists despite suppression attempts and interferes with daily functioning (American Psychiatric Association, 2013). However, the diagnostic threshold relies more on duration and functional impairment than on symptom nature (Crocq, 2017). This raises the question of whether the threshold marks not pathology but a level of symptom visibility sufficient for measurement.

Moreover, consensus is lacking regarding tools for measuring general(ised) anxiety. One widely used instrument for GAD is the GAD-7 questionnaire. Although aligned with DSM-5 criteria, it assesses symptoms over only the past 2 weeks and omits key features like muscle tension or unstoppable worry (Spitzer et al., 2006). Meanwhile, the STAI-T, though intended to measure trait anxiety, includes items reflecting depressive states and may mix anxiety with general negative affect (Davey et al., 2022). Despite these differences, these two scales strongly correlate (Bentley et al., 2021; Doi et al., 2018) and are often used interchangeably as indicators of a shared tendency to worry (Likhanov et al., 2026).

From the neurobiological perspective, both high TA and GAD are associated with attentional bias toward threat and difficulty disengaging from negative stimuli (Bar-Haim et al., 2007). Studies reveal shared disruptions in top-down regulation: individuals high in TA show reduced prefrontal control over amygdala reactivity (Bishop, 2009), while GAD patients exhibit altered connectivity in the anterior cingulate and medial prefrontal cortices, regions linked to emotion regulation (Cui et al., 2019; Xing et al., 2017). This convergence supports a continuum view. Longitudinal data indicate that children with high TA are at increased risk for developing GAD (Ma et al., 2017), and genetic studies suggest shared heritability between TA, GAD, and other internalizing disorders (Kendler and Myers, 2014). Rather than viewing TA as a trait and GAD as a pathology, it may be more accurate to see them as points along a continuum, where trait anxiety represents latent risk that, under stress or without protective factors, can crystallize into a clinical disorder.

### 1.2 Domain-specific anxieties: more than just trait anxiety

**Figure 1:**
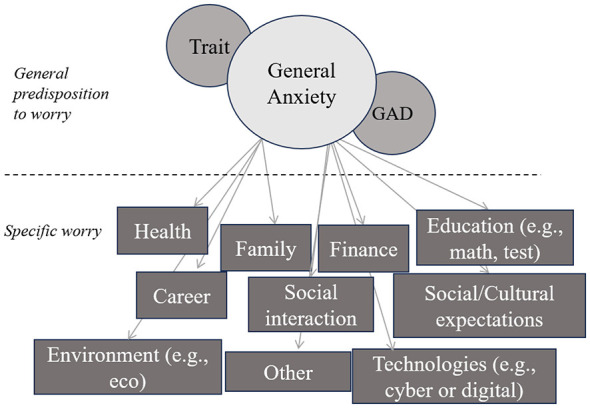
Dual structure of anxiety.

While trait anxiety reflects a general predisposition to worry about any negative outcome, specific anxieties are typically described as worry tied to concrete stimuli or situations. One well-studied example is math anxiety, associated with poor math performance (Barroso et al., 2021) even after controlling for TA (Hill et al., 2016). Similarly spatial anxiety is linked to lower navigation and mental rotation performance (Geer et al., 2024; Lawton, 1994). Social anxiety, though moderately correlated with trait anxiety (*r* = 0.40–0.50, indicating shared variance about 16%−25%; Martínez Valera et al., 2024), is associated with specific neural responses to social-evaluative threat not observed in GAD (Wong et al., 2020). Studies also show that specific anxieties moderately to highly correlate with each other and with trait anxiety (*r* = 0.30–0.70, indicating shared variance up to 50%; Likhanov et al., 2026). Current literature identifies numerous specific anxieties such as test anxiety (Spielberger, 1980), health anxiety (Anniko et al., 2019), foreign language anxiety (Alrabai, 2014), and eco-anxiety (Orrù and Mannarini, 2024). Notably, most overlap with fundamental life domains like health, finance, or career (Larsen et al., 2023; Scherer and Tannenbaum, 1986; Tsukerman et al., 2020). These findings challenge fully independent anxiety models and instead suggest a dual-structure approach, where general anxiety coexists with domain-specific components and explains additional, context-dependent variance (Figure 1).

A recent adolescent study found that while a general anxiety factor underlies multiple self-report measures, domain-specific anxieties predict lower academic performance in corresponding subjects (e.g., math anxiety with math grades, spatial anxiety with STEM outcomes). Interestingly, after controlling for other anxiety types, worry remained associated with academic performance across all domains (β = 0.12; Likhanov et al., 2026). These results were replicated in adults: while the general anxiety factor was weak or absent, domain-specific anxieties (math, social, spatial) selectively impaired performance in corresponding cognitive tasks, supporting the idea that the functional impact of anxiety depends on alignment between worry content and task domain (Alenina et al., 2025). A network analysis of 150 everyday worries also highlighted the role of actual worries (frequency and intensity), with family and health emerging as central hubs, while technology-related anxieties showed heightened emotional intensity likely reflecting contemporary societal pressures (Alenina and Kosonogov, 2025). Additionally, participants with high TA exhibited a fragmented worry structure, suggesting that anxiety severity may reshape not only symptom load but also the organization of worry itself.

In sum, anxiety is neither purely general nor entirely specific but operates on a dual-structure model. A broad predisposition to worry (TA) sets the baseline threat sensitivity, but functional impact of anxiety arises primarily through domain-specific manifestations that selectively impair, or potentially enhance, performance in specific contexts. For example, test anxiety is typically observed in student samples (Brady et al., 2018), whereas health and death anxiety studies focus mostly on older adults (El-Gabalawy et al., 2013). Critically, specific anxieties retain unique predictive power beyond TA, underscoring the need for further research.

### 1.3 Methodological and conceptual gaps

While conceptual boundaries between general and specific anxiety remain debated, existing psychological tools assess manifestations in isolation. No current questionnaire simultaneously captures general anxiety (both clinical and non-clinical) and key domain-specific forms. Clinically oriented instruments such as the GAD-7 (Spitzer et al., 2006) or Anxiety Symptoms Questionnaire (Baker et al., 2019) focus on DSM-aligned symptoms (e.g., excessive worry, panic, social avoidance). Similarly, the ANX-8 (Rossiter, 2022) or Anxiety Disorder Diagnostic Questionnaire (ADD; Norton and Robinson, 2010) distinguishes anxiety disorders like OCD and social anxiety but does not link them to life-domain worries such as academic performance. While these tools offer broad screening, they lack integration of trait severity with contemporary domain-specific concerns within a single framework. Such a scoring approach allows researchers to test whether there are any domain-specific worries beyond the general factor. Meanwhile, domain-specific scales, such as the Abbreviated Math Anxiety Scale (AMAS; Hopko et al., 2003) or the Spatial Anxiety Scale (Lawton, 1994), are often developed in isolation, with little integration into broader anxiety frameworks. Crucially, no widely used instrument integrates general trait anxiety (e.g., STAI-T), clinical severity (e.g., GAD-7), and a comprehensive set of content-specific worries (e.g., health, technology, family, eco-anxiety) within a single framework.

This fragmentation limits both research and practice. When general and specific anxieties are measured separately, distinguishing shared from unique variance becomes difficult. For instance, studying math anxiety may require controlling for both trait and clinical anxiety. Moreover, the absence of unified tools impedes empirical testing of dual-structure models and the interplay between general and specific worries (Epskamp and Isvoranu, 2022; Reise et al., 2013). Beyond factor-analytic approaches, network analysis (Alenina and Kosonogov, 2025) represents anxiety as a system of mutually reinforcing nodes (specific and general worries) and clustering into hubs (e.g., health-family worries). Unlike latent variable models, this approach does not assume a general factor causes symptoms; rather, it models direct interactions between worry domains. This complements the hierarchical framework by identifying central hubs that may drive co-activation, offering a statistical alternative for fitting multidimensional anxiety data.

Developing a unified tool is especially critical in educational psychology, where anxiety can influence career choices, for example, math anxiety leads students to avoid STEM regardless of ability (Daker et al., 2021). Similarly, emerging constructs like digital-life anxieties (e.g., data privacy; Pink et al., 2018) or ecological concerns (eco-anxiety; Orrù and Mannarini, 2024) are not captured by clinical instruments, despite evidence of their psychological burden. Some multidimensional tools exist but focus mainly on clinical aspects. For example, the Penn State Worry Questionnaire (Meyer et al., 1990) measures pathological worry without differentiating content domains. The Anxiety Sensitivity Index (Peterson, 1994) assesses fear of anxiety-related sensations but emphasizes somatic concerns without linking them to broader contexts. Overall, a comprehensive tool could have a modular assessment architecture: a core module measuring general worry proneness and its domain-specific modules selected based on context with the computerized adaptive testing (CAT) approach which could dynamically select items to maximize precision while minimizing respondent burden. This structure addresses the bandwidth-fidelity trade-off by maintaining reliability within short modules and without excessive length. Each module could undergo separate psychometric validation, with measurement invariance tested across contexts to ensure comparability of scores. Such a scoring approach allows researchers to test whether there are any domain-specific worries beyond the general factor.

## 2 Discussion

In summary, anxiety cannot be viewed as a unitary construct. Instead, it functions as a dual-structure hierarchical system: general anxiety forms a continuum from milder traits to severe, prolonged GAD symptoms, while domain-specific anxieties emerge in particular contexts (e.g., math anxiety in educational settings). Specifically, general anxiety might account for the positive covariation among domain-specific factors, as orthogonal residuals that retain unique variance beyond the general factor. This positions general anxiety as a higher-order factor driving covariation, rather than a distinct moderator. Consequently, instrument scoring must partition variance into general and orthogonal specific residuals to isolate context-dependent impairment. These specific forms are not mere byproducts of high trait anxiety; they carry unique predictive power for performance, avoidance, and distress-even in high-functioning individuals.

Yet this complexity has contributed to the lack of comprehensive, integrative measurement tools. Existing instruments are fragmented: they capture either general, clinical, or specific characteristics in isolation, largely ignoring how domain-specific anxieties relate to a broader worry predisposition. An integrative tool (e.g., based on the CAT) could assess the intensity (including perceived uncontrollability), frequency, and specificity of worry. Items taking into account uncontrollability could be adapted from established formulations (e.g., GAD-7; Spitzer et al., 2006), while acknowledging that current diagnostic thresholds rely more on duration and functional impairment than on symptom content alone (Crocq, 2017). This approach would help operationalize a distinction between manageable worry and clinical manifestations. Such a design could mitigate participant fatigue, ensuring that the data quality remains high even when assessing multiple domains. Ultimately, clarifying how general and specific worries jointly contribute to impairment may provide more nuanced, context-sensitive thresholds for clinical significance, particularly in educational or occupational settings where anxiety often manifests as a performance barrier rather than a formal disorder.

Future studies should examine multiple domain-specific anxieties alongside trait anxiety using longitudinal designs to track their development and interactions across the lifespan. Studies of brain dynamics could clarify whether different anxiety domains share underlying neural networks. Methodologically, there is a clear need for an integrative tool that assesses both trait and specific anxiety simultaneously. Such an instrument would better differentiate between general and specific components and enable network analyses to reveal how anxieties co-activate and reinforce one another.

To conclude, the current fragmentation of anxiety constructs reflects both the inherent complexity of emotion and our rapidly changing world. The way forward lies not in choosing between general or specific models but in integrating them into a coherent framework that accounts for both the universality of worry and its rich contextual variation. Developing a new comprehensive tool and rethinking outdated categories will help advance the field.
